# “Indirect development” increases reproductive plasticity and contributes to the success of scyphozoan jellyfish in the oceans

**DOI:** 10.1038/s41598-021-98171-w

**Published:** 2021-09-20

**Authors:** Isabella D’Ambra, Louise Merquiol, William M. Graham, John H. Costello

**Affiliations:** 1 Department of Integrative Marine Ecology, Stazione Zoologica Anton Dohrn, Villa Comunale, 80121 Napoli, Italy; 2 Florida Institute of Oceanography, 830 1st Street S. MSL 128D, St. Petersburg, FL 33701 USA; 3 Biology Department, Providence College, Providence, RI 02918 USA

**Keywords:** Ecology, Evolution, Environmental sciences, Ocean sciences

## Abstract

Ecologists and evolutionary biologists have been looking for the key(s) to the success of scyphomedusae through their long evolutionary history in multiple habitats. Their ability to generate young medusae (ephyrae) via two distinct reproductive strategies, strobilation or direct development from planula into ephyra without a polyp stage, has been a potential explanation. In addition to these reproductive modes, here we provide evidence of a third ephyral production which has been rarely observed and often confused with direct development from planula into ephyra. Planulae of *Aurelia relicta* Scorrano et al. 2017 and *Cotylorhiza tuberculata* (Macri 1778) settled and formed fully-grown polyps which transformed into ephyrae within several days. In distinction to monodisk strobilation, the basal polyp of indirect development was merely a non-tentaculate stalk that dissolved shortly after detachment of the ephyra. We provide a fully detailed description of this variant that increases reproductive plasticity within scyphozoan life cycles and is different than either true direct development or the monodisk strobilation. Our observations of this pattern in co-occurrence with mono- and polydisk strobilation in *Aurelia* spp. suggest that this reproductive mode may be crucial for the survival of some scyphozoan populations within the frame of a bet-hedging strategy and contribute to their long evolutionary success throughout the varied conditions of past and future oceans.

## Introduction

Cnidaria—Anthozoa (stony and soft corals and sea anemones) and Medusozoa (Hydrozoa, Scyphozoa and Cubozoa)—display a high degree of adaptive radiation and have colonised almost all marine as well as some brackish and freshwater habitats. Cnidarian colonisations started near the initial stages of animal evolution on Earth. Cnidarians branched off early within metazoan evolution and differentiated as key predators during the Middle Cambrian. Four classes (Anthozoa, Hydrozoa, Scyphozoa, Cubozoa) have been identified within the Ediacaran fauna^1^, although interpretation of Ediacaran fossils as medusae^2–4^ remains contentious^5^. The analysis of the genome within and across the classes of cnidarians indicates that Anthozoa have a circular DNA, while Medusozoa possess a linear genome, which suggests that the Anthozoa preceded the Medusozoa^6^. However, while this finding clarifies the phylogeny of the phylum, the key(s) to such adaptive success have not been identified. Here, we describe a novel, “indirect development” reproductive strategy that may contribute to the success of scyphozoans across time and habitats within the frame of a reproductive plasticity which increases the fitness of this group to diverse environmental conditions.

The diverse circumstances of this long evolutionary period have required cnidarians to tolerate and adapt to ever-changing oceanic conditions. However, contemporary rates of environmental change raise a number of pressing questions linked to survivorship. For example, coral reefs are threatened by a host of stressors including temperature increase and ocean acidification^7^. Medusozoan pelagic stages, particularly hydromedusae and scyphomedusae, gave rise to mass occurrences in the past, as suggested by marine fossil deposits from the Late Cambrian^8^. At present, hydro- and scyphomedusae still often give rise to sudden and unpredictable blooms and outbreaks, whose frequency and intensity may be increasing worldwide^9–11^. The apparent increase of medusozoans has been attributed to anthropogenic stressors and degradation of the marine environment (overfishing, eutrophication, increased habitat suitability for polyps)^12–15^. Nevertheless, the mechanisms behind differing responses by anthozoans and medusozoans to changing environmental conditions remain obscure.

Variations in reproductive strategies of anthozoans and medusozoans may explain, at least in part, their diverse response to changing environmental conditions. Anthozoans display a single life stage, the polyp, which lives isolated or in colonies. Conversely, most hydro- and scyphozoans have a metagenic life cycle, which includes a transition from the benthic polyp (like anthozoans) to a free-swimming pelagic medusa stage^16–18^. Both hydro- and scyphozoan life cycles exhibit a high degree of plasticity, with exceptions to the general patterns. For example, the hydrozoans *Laodicea undulata* (Forbes and Goodsir 1853) and *Turritopsis* spp. are capable of “reversing” their life cycle by regressing from medusa into polyp when conditions dictate^19,20^. Within scyphozoans, the medusae of *Aurelia* sp. are able to reverse their life cycle by regenerating a polyp from a medusa^21^. Overall, the reproductive patterns of scyphozoans appear to present a greater spectrum of alternatives than those of other cnidarians.

The production of young medusae (ephyrae) in scyphozoans occurs in two modes: strobilation (either mono- or polydisk) and direct development from planula into ephyra without a polyp stage^16^. Strobilation is an asexual reproductive strategy by which the polyp generates one (monodisk) or up to more than 15 ephyrae (polydisk)^16^. Within scyphozoans, only *Pelagia noctiluca* (Forsskål 1775) and *Periphylla periphylla* (Péron & Lesueur 1810) are known to have a direct development^22,23^. Reviews of the life history traits and abiotic variables potentially driving mass occurrence of scyphomedusae highlight the strobilation phase as a key factor in outbreak formation because of the large number of ephyrae that each single polyp can release^16,24^. Laboratory experiments indicated that temperature, light and food availability affect the number and size of produced ephyrae^16^. However, the small size of polyps and ephyrae along with the difficulty of polyp detection in their natural habitat has constrained their study in situ and observations in laboratory have not been corroborated by field data.

Variations within the scyphozoan reproductive cycle may be widespread and affect population survival. This is particularly true for strobilation strategies^25,26^. However, the clarity of scyphozoan reproduction patterns is highly variable in nomenclature surrounding reproduction. For example, polyps of *Aurelia* spp. usually show a polydisk strobilation^16^. However, monodisk strobilation has been reported to alternate with polydisk strobilation within the same population of *Aurelia aurita* (Linnaeus 1758) in Gullmar Fjord (Sweden) depending on higher or lower abundance of prey, respectively^27^. Studies from Japan reported the production of a single ephyra with loss of the basal polyp after detachment of the ephyra^28–30^, which was called “direct development”^30^. Recently, Suzuki et al.^31^ indicated that *Aurelia coerulea* von Lendenfeld 1884 underwent “direct development” (i.e. planulae transformed directly into ephyrae) from December to May, while the same population showed a metagenic life cycle, which included a polyp stage performing a polydisk strobilation during summer months. However, both a picture and a drawing clearly show the ephyra attached to a substrate by a thin stalk, which appears similar to the ephyra production mode described by Yasuda^30^. The lack of a fully detailed description of this unusual reproductive strategy and the name “direct development” likely generated confusion with the true direct development from planula into ephyra without a polyp stage as observed in *P. noctiluca* and *P. periphylla*^23,32^.

In this study, we provide a detailed description of an atypical ephyral production pattern in which the polyp stage is unable to regenerate after ephyra detachment in the scyphozoans. This occurs in both *Aurelia relicta* Scorrano et al. 2017 from Mljet (Croatia) and *Cotylorhiza tuberculata* (Macri 1778) from the bay of Pozzuoli (southern Italy). This reproductive strategy appears very similar to the ephyral production described by Yasuda and Suzuki et al.^30,31^. To avoid confusion in the future, we suggest defining this pattern as “indirect development” to clearly differentiate it from both monodisk strobilation and classical direct development. Given that this reproductive pattern has been observed across *Aurelia* spp. populations in different habitats and co-occurring with both mono- and polydisk strobilation within a single population, we suggest it may be part of a reproductive portfolio within a bet-hedging strategy to enhance reproductive success and therefore we propose to include it within *Aurelia* spp. life cycle (Fig. 3). Indeed, the observation of this pattern in two taxonomically and evolutionary distant species (*Aurelia relicta* and *Cotylorhiza tuberculata*) suggests that indirect development may not be restricted to *Aurelia* spp. and is, instead, a component of an expanding spectrum of reproductive variations within the Scyphozoa. We discuss whether such nuances may contribute to their adaptive flexibility in diverse environments and favour their survival in future ocean scenarios.

## Materials and methods

### Sampling

*Aurelia relicta* adult medusae were collected in the Big Lake of Mljet National Park (Croatia) by SCUBA divers in May 2004 as part of the research activities within the project “Meduza”, which was developed to examine the population dynamics of the scyphozoan in the semi-enclosed ecosystem^33^. Medusae were placed individually in plastic bags filled with seawater and transported to the laboratory. A single female *Cotylorhiza tuberculata* was collected using a dip net in the bay of Pozzuoli, located in the north-eastern part of the Gulf of Naples (Italy) during October 2019 within the sampling activities of the project “ABBaCo”^34^. The medusa was placed in a plastic bucket filled with seawater and transported to the laboratory.

### Rearing of scyphozoan polyps

Planulae were collected from both *A. relicta* and *C. tuberculata* medusae using glass pipettes. Randomly selected planulae were placed into 6-well culture plates (10 planulae per well for both scyphozoan species) filled with filtered seawater and maintained at constant temperature (18–20 °C) and salinity (38) throughout their development. When polyps fully developed and had tentacles, they were fed ad libitum twice per week with newly-hatched *Artemia* sp. brine shrimp nauplii. Nauplii were removed from each well 12 h after being introduced to avoid fouling of the wells.

## Results

**Figure 1:**
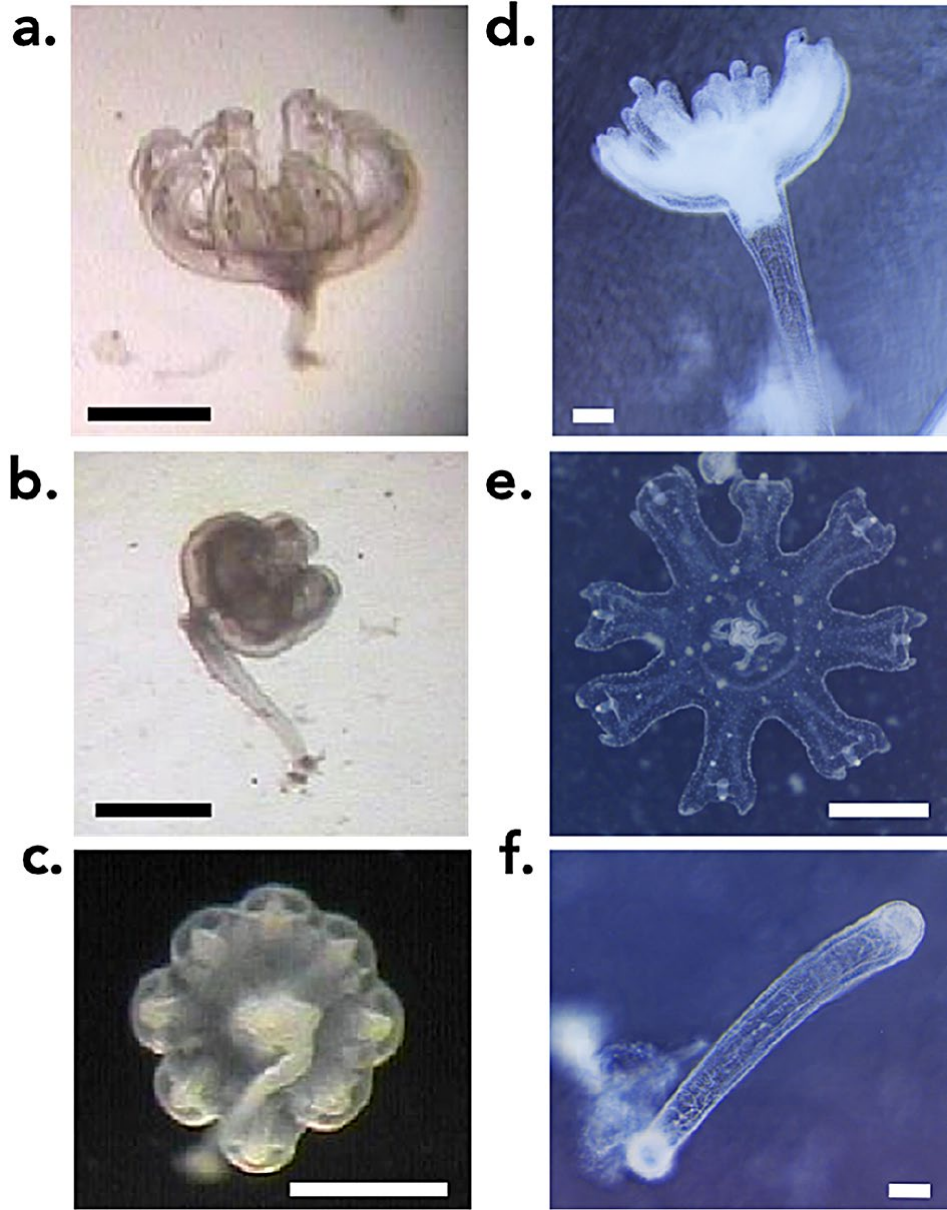
Indirect development sequence in (**a**–**c**) *Aurelia relicta* Scorrano et al. 2017 from Mljet (Croatia); (**d**–**f**) *Cotylorhiza tuberculata* (Macri 1778) from the bay of Pozzuoli (Italy). Scale bars = 500 µm.

The “indirect development” occurred with similar features in both *Aurelia relicta* and *Cotylorhiza tuberculata* (Fig. 1). Planulae (n = 10 per well) placed in each well of 6-well culture plates settled within 3–6 days and developed into fully-grown polyps (72 polyps out of 120 planulae for *Aurelia relicta* and 180 out of 240 for *Cotylorhiza tuberculata*, Table 1) within the following 3–6 days. Within 2 months, 8% and 13% of the fully-grown polyps for *A. relicta* and *C. tuberculata*, respectively, underwent the transition into ephyra in separate wells for *A. relicta*, while we observed up to three indirect developments in the same well for *C. tuberculata* (Table 1). Tentacles were absorbed, the calyx widened and lappets formed so that the ephyra shape became almost complete (Fig. 1a,d). The following day, the lappets and the rhopalia were fully formed (Fig. 1b) and the ephyra began to pulse. Within the third day, the ephyra was freely swimming in the well (Fig. 1c,e). In two out of 29 ephyrae, the stalk remained attached to the ephyra and then was lost (Fig. 1c), but it detached quickly in most (n = 27) cases (Fig. 1e,f). In all indirect developments observed (n = 29), the stalk did not regenerate into a new polyp (Fig. 1f) and dissolved shortly after detachment of the ephyra.

**Table 1:** The number of planulae collected from adult scyphomedusae, full-grown polyps, indirect developments, mono- and polydisk strobilations observed in *Aurelia relicta* from Mljet (Croatia) in 2004 and *Cotylorhiza tuberculata* from the bay of Pozzuoli (southern Italy) in 2019.

Species	Planulae	Polyps	Indirect development	Monodisk strobilation	Polydisk strobilation
*Aurelia relicta*	120	72	6	8	58
*Cotylorhiza tuberculata*	240	180	23	–	–

Mono- and polydisk strobilations (8 and 58 polyps, respectively, Table 1) were observed for *A. relicta* only at the same time as indirect development. We observed mono- and polydisk strobilations co-occurring in the same wells and polydisk strobilation co-occurring in the same wells as indirect development. Most polyps (80%) underwent polydisk strobilation, while the number of polyps reproducing via monodisk strobilation and indirect development was lower (11% and 8%, respectively, Table 1).

**Figure 2:**
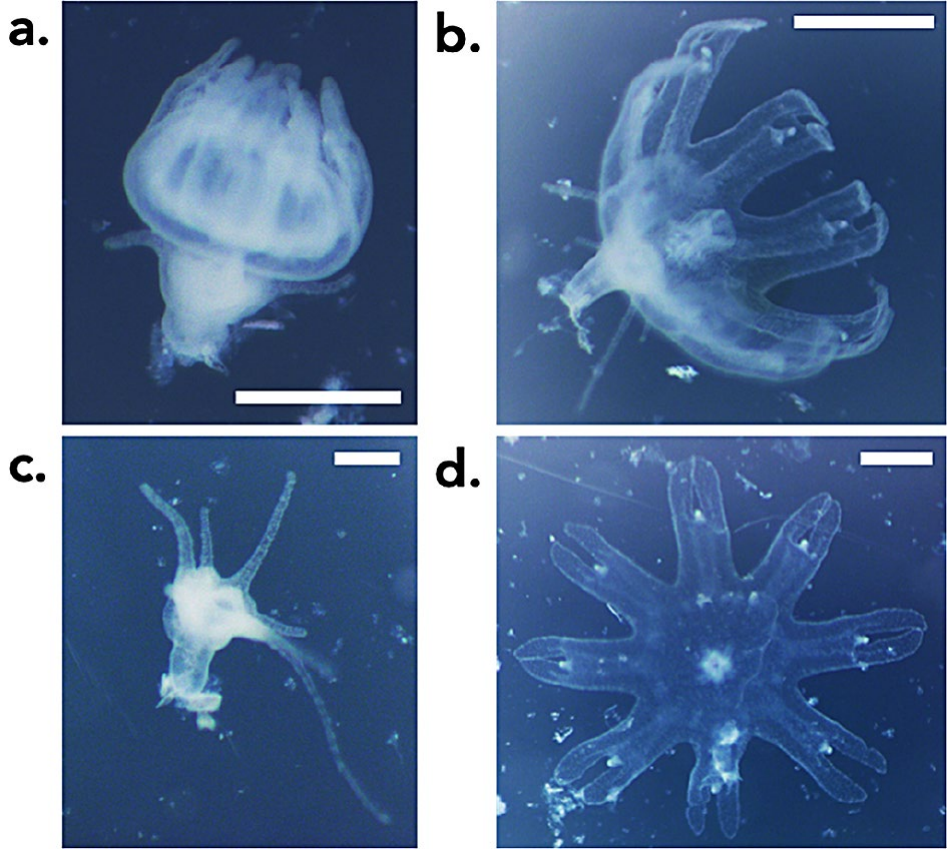
Monodisk strobilation of *Aurelia relicta* Scorrano et al. 2017 from Mljet (Croatia) observed in co-occurrence with indirect development. Scale bars = 500 µm.

Polydisk strobilation occurred following the same patterns described in previous studies^35^ and therefore is not shown in the present one. We provide a description of monodisk strobilation to highlight the differences from indirect development. In distinction to indirect development, the termination of monodisk strobilation was characterized by tentacle retention of the basal polyp (Fig. 2b). Monodisk polyps did not degenerate to a stalk, but remained attached to the wall of the well and within few hours regenerated a full complement of tentacles (Fig. 2c) following ephyra detachment (Fig. 2d). Although the reproductive pattern observed in this study maintains some similarities to monodisk strobilation, we note the distinction of a non-viable polyp which lacks the ability to regenerate tentacles and term this pattern “indirect development” to reflect the transformation of the polyp into an ephyra.

The patterns of the indirect development were very similar between the two scyphozoan species, but not the frequency of occurrence among planulae (Table 1). Approximately 5% of the planulae collected from *A. relicta* and about 10% of those from *C. tuberculata* underwent the indirect development.

## Discussion

Modern oceans are inhabited by an evolutionary mix of recently derived and archaically successful organisms. Why would some early (i.e., pre-Cambrian) groups like the cnidarian medusae persist relatively unchanged over biological time, while other taxa evolve, modify, and go extinct? We suggest that ancient life-history strategies of early metazoans evolved buffers against extreme fluctuations in the environment (salinity, temperature, food, etc.). This same life-history bet-hedging strategy likely allows the cnidarian medusae to flourish in modern oceans despite human-mediated climate alterations, radical changes in food-web structure and other coastal ocean structural changes. This is exemplified by recent observations of unusual developmental reversals of young medusae as they transform directly back into the sessile stage or polyps transforming into cysts and then excyst and reproduce asexually during unfavourable seasonal conditions^25,26^. The plasticity permitted by this spectrum of responses provides an evolutionary buffer against sudden and unexpected environmental shifts^21^ that might make a single strategy unfavourable across a range of conditions.

**Figure 3:**
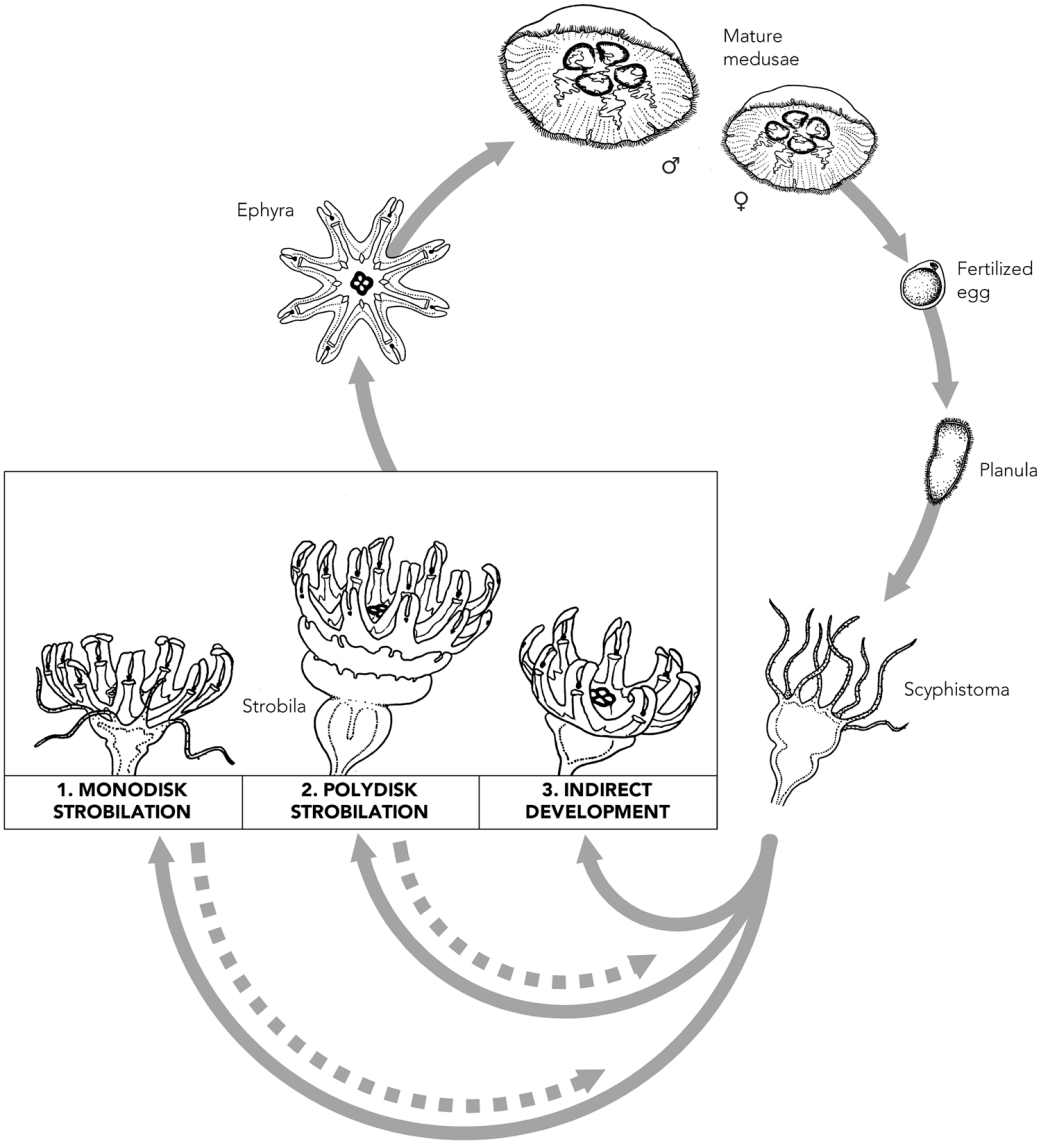
Revised life cycle of *Aurelia* sp. with the addition of the indirect development as a reproductive strategy to produce ephyrae in addition to mono- and polydisk strobilations (drawing by Louise Merquiol).

The indirect development described in the present study falls within these variations in reproductive patterns. Because the atypical reproductive mode (Fig. 1) was observed only in *Aurelia* spp. from Japan^28–30^ until now and it has never been included within the reproductive types of *Aurelia* spp.^16,17^. It was likely considered a rare reproductive mode restricted to limited populations of specific areas. Yet the definition of this reproductive pattern as “direct development”^30^ generated confusion with the true direct development as observed in *Pelagia noctiluca* and *Periphylla periphylla*, where the polyp stage is totally absent^22,23^. A recent report^31^ may be an example of this potential confusion, where the authors indicated that > 90% of the *Aurelia coerulea* ephyrae found during December to May in Maizuro Bay (Japan) were produced via “direct development”, while a regular metagenic life cycle with a polyp stage reproducing via polydisk strobilation was observed during the summer months. However, pictures show the same pattern we describe in the present study, with ephyrae anchored to a substrate by a thin stalk, similar to the last phase of indirect development just before ephyra detachment and loss of the stalk (Fig. 1b). The similarity between the two reproductive modes in two *Aurelia* species from distant areas suggests that indirect development may not be a rare reproductive pattern as previously considered^28,29,36^. Conversely, it may play a critical role in ensuring the survival of some species or populations. Based on these considerations, we suggest inclusion of indirect development as a third reproductive pathway within ephyral generation processes in *Aurelia* spp. at present (Fig. 3), but potentially in other scyphozoans as well.

The sequence of indirect development stages (Fig. 1) is distinctly different from the direct development observed in *Pelagia noctiluca* and *Periphylla periphylla*^22,23^. During the indirect development of *A. relicta* and *C. tuberculata*, planulae settled and developed into a fully-grown polyp, followed by a rapid transformation into ephyra (Fig. 1a,d). Compared to monodisk strobilation (Fig. 2), the distinguishing characteristic of indirect development is that the basal polyp is unable to regenerate and dies (Fig. 1f). The process leads to the production of a single ephyra with a loss of the benthic stage. This latter trait is important from a population perspective because the polyp is unable to generate new ephyrae via subsequent strobilations and cannot contribute to further population recruitment.

Although the basal polyp is lost and therefore ephyral production stops, indirect development may still have an advantage in terms of success for a species or a population. The fact that we observed monodisk and polydisk strobilations at the same time as indirect development within the same population of *A. relicta* polyps suggests that different reproductive modes may co-exist within an overarching bet-hedging strategy. By having multiple reproductive patterns, a species or population may maximize reproductive success, balancing the investment in reproduction and the costs of reproductive efforts, as it has been demonstrated for organisms belonging to different groups across trophic levels^37^.

Within scyphozoans, *Aurelia* spp. have shown to adapt to a variety of environments. Based on molecular and phylogenetic analyses, this genus appears to be one of the most ancient within scyphozoans, with ancestors likely swimming in the Tethys ocean and then undergoing a wide adaptive radiation across most seas formed after the disappearance of the ancestral unique ocean^38^. In Gullmar Fjord, *Aurelia aurita* polyps alternated mono- and polydisk strobilations according to food availability^27^. In Maizuro Bay (Japan), *A. coerulea* alternated indirect development during winter to polydisk strobilation in summer^31^. Our observation of a co-occurrence of multiple reproductive modes of *A. relicta* in the present study along with the observations by Japanese authors^29–31^ suggest that multiple reproductive strategies may be a common trade-off within *Aurelia* spp. and explain, at least in part, the adaptive success of this genus across time and habitats.

For scyphozoans, the effect of environmental forcing on multiple reproductive strategies is difficult to define based on our observations and the information available in literature. Additionally, very little is known about the natural habitat of polyps of *A. relicta* and *C. tuberculata* in Mljet lake and the Bay of Pozzuoli, respectively. Salinity was kept constant (38) throughout the rearing period for both species, and this value is likely the same that polyps experience in their natural habitat, considered this is the average value both in Mljet lake^39^ and the bay of Pozzuoli^34^.

As for temperature, *Aurelia* spp. polyps usually perform a polydisk strobilation at a wide range of temperatures (reviewed by^16^), while the temperature at which *Aurelia aurita* polyps were observed to do a monodisk strobilation in Gullmar Fjord was not reported^27^. The temperature we kept our polyps (18–20 °C) falls within the range of temperature at which polydisk strobilation was observed^16^. Indeed, we observed both mono- and polydisk strobilations in separate and the same wells as indirect development. Little information is available for *C. tuberculata*^40,41^. However, as for *A. relicta*, our temperature range (18–20 °C) is the same as the range at which monodisk strobilation was observed for this species^40,41^. Therefore, a clear influence of temperature cannot be inferred to initiate the atypical reproductive pattern described in the present study, at least with the data and information in our possession.

Food availability has been shown to affect the number of ephyrae generated during polydisk strobilation^16^. During high prey density, polyps are able to produce more ephyrae per individual polyp than during low prey availability (reviewed by^16^). The only case of monodisk strobilation in *Aurelia aurita* from Gullmar Fjord was correlated with low prey density, while polyps reproduced via polydisk strobilation during high prey availability^27^. Field observations of indirect development (defined in that study as “direct development”^30^) peaked (> 90%) from December to May, during low zooplankton biomass^31^: Polyps of both species in the present study were fed ad libitum and likely did not experience limited prey availability. Although local or temporary inability to feed on an appropriate amount of prey may have occurred for some polyps despite the presence of abundant prey in the wells, the fact that indirect development occurred in the same wells where other polyps underwent polydisk strobilation suggests that, at least for *Aurelia relicta*, food limitation did not occur. The high availability of food potentially allowed polyps to reproduce using a spectrum of strategies. However, the limited observations of indirect development both in laboratory and in situ and the information available about other reproductive strategies in scyphozoans do not allow define the effect of environmental factors on reproductive strategies in scyphozoans, which remain a subject requiring more extensive research.

Planulae collected from *C. tuberculata* did not contain the endosymbiotic dinoflagellates which are usually found within the mesoglea of the adult specimens^42^. According to a recent review, polyp infection occurs in the environment after planulae have settled^43^. Therefore, our polyps kept in filtered sea water could not capture the endosymbionts from the surrounding environment and were aposymbiotic. Similarly to other zooxanthellate scyphozoans, *C. tuberculata* displays a monodisk strobilation and includes in its metabolic budget the compounds photosynthesized by the symbionts^16,43^. The lack of the compounds photosynthesized by the endosymbiotic hosts may have favoured the indirect development in comparison to monodisk strobilation, since we did not observe any monodisk strobilation in co-occurrence with indirect development. The scarcity of information about the mechanisms behind strobilation in zooxanthellate scyphozoans and the limited number of observations of indirect development in the present study do not allow us to draw conclusions, but stimulate future laboratory and in situ observations to shed light on the reproductive plasticity of this particular group within scyphozoans.

Scyphozoan polyps show a higher degree of reproductive plasticity than anthozoan corals. While scyphozoan polyps without endosymbionts reproduced, most anthozoan polyps die after losing their symbionts^44^. The different response to loss of symbionts may be an advantage for scyphozoan polyps that may be able to survive after losing their symbionts due to unfavourable future conditions in the oceans (e.g. increased temperature and/or lower pH).

## Conclusions

Indirect development has been long overlooked within the context of scyphozoan reproductive strategies. This may largely be due to the fact that is an atypical reproductive mode where a short-lived, fully-grown polyp produces a single ephyra but is unable to regenerate and reproduce again. Based on previous reports, indirect development appeared to be either genus-specific or restricted to a very limited geographic area. The lack of a detailed description of this reproductive strategy and the definition “direct development”^30^ likely has contributed to underestimation of its occurrence and generated confusion with true direct development described for *Pelagia noctiluca* and *Periphylla periphylla*. Alternatively, we describe indirect development in two distantly related species, indicating that the process may be more common across scyphozoan taxa than expected. Although full understanding about the conditions that regulate indirect development will require rigorous testing, our preliminary observations suggest that this pattern may be part of a bet-hedging strategy which allows scyphozoans to enhance overall survival chances. Flexible life history patterns have likely influenced the broad success of scyphozoans across time and space. Indirect development may play an important role in the varied life history portfolio contributing to the success of scyphozoans during the rapid changes occurring in contemporary oceans.
